# Assessment of the risk of permanent stoma after low anterior resection in rectal cancer patients

**DOI:** 10.1186/s12957-020-01979-5

**Published:** 2020-08-14

**Authors:** Marcin Zeman, Marek Czarnecki, Andrzej Chmielarz, Adam Idasiak, Maciej Grajek, Agnieszka Czarniecka

**Affiliations:** 1 The Oncologic and Reconstructive Surgery Clinic, Maria Sklodowska-Curie National Research Institute of Oncology, Gliwice Branch, Wybrzeze Armii Krajowej 15, 44-100 Gliwice, Poland; 2 II Clinic of Radiotherapy and Chemotherapy, Maria Sklodowska-Curie National Research Institute of Oncology, Gliwice Branch, Wybrzeze Armii Krajowej 15, 44-100 Gliwice, Poland

**Keywords:** Low anterior resection, Rectal cancer, Loop ileostomy, Permanent stoma, Anastomotic leakage, Fibrinogen

## Abstract

**Background:**

One of the most severe complications of low anterior rectal resection is anastomotic leakage (AL). The creation of a loop ileostomy (LI) reduces the prevalence of AL requiring surgical intervention. However, up to one-third of temporary stomas may never be closed.

The first aim of the study was to perform a retrospective assessment of the impact of LI on the risk of permanent stoma (PS) and symptomatic AL. The second aim of the study was to assess preoperative PS risk factors in patients with LI.

**Methods:**

A total of 286 consecutive patients who underwent low anterior rectal resection were subjected to retrospective analysis. In 101 (35.3%) patients, diverting LI was performed due to low anastomosis, while in the remaining 185 (64.7%) patients, no ileostomy was performed. LIs were reversed after adjuvant treatment. Analyses of the effect of LI on symptomatic AL and PS were performed. Among the potential risk factors for PS, clinical factors and the values of selected peripheral blood parameters were analysed.

**Results:**

PS occurred in 37.6% and 21.1% of the patients with LI and without LI, respectively (*p* < 0.01). Symptomatic ALs were significantly more common in patients without LI. In this group, symptomatic ALs occurred in 23.8% of patients, while in the LI group, they occurred in 5% of patients (*p* < 0.001). In the LI group, the only significant risk factor for PS in the multivariate analysis was preoperative plasma fibrinogen concentration (OR = 1.007, 97.5% CI 1.002–1.013, *p* = 0.013).

**Conclusions:**

Although protective LI may reduce the incidence of symptomatic AL, it can be related to a higher risk of PS in this group of patients. The preoperative plasma fibrinogen concentration can be a risk factor for PS in LI patients and may be a useful variable in decision-making models.

## Background

In rectal cancer surgery, there is a noticeable tendency to widen the indications for sphincter-sparing procedures. The most serious complication of these procedures is anastomotic leakage (AL), whose prevalence in low anterior rectal resections (LAR) can reach even 30% [1]. Although the effect of loop ileostomy (LI) on reducing the symptomatic prevalence of AL has been demonstrated, the prevalence of permanent stoma (PS) after LAR with LI may exceed 30%, and in elderly patients, this rate can even reach 50% [2, 3].

In addition, given the possible complications associated with the presence of ileostomy, such as electrolyte disturbances, renal dysfunction, and the risk of surgical complications associated with the ileostomy itself and ileostomy closure procedures, it is necessary to define the group of patients who actually benefit from elective diversion [4–6]. The rate of non-closure of the stoma is difficult to compare due to different criteria adopted by authors, including different latest expected time to closure which ranges from 9 to 36 months. In different centres, patients are qualified for the closure of LI before, at the time of or after adjuvant treatment. The presence of PS may be caused by no scheduling for closure of LI and the necessity for stoma recreation after the previous closure procedure [7].

The risk factors for PS include advanced age, advanced stage of the disease, narrow radial margin, colorectal AL and adjuvant therapy [8, 9]. However, some of these factors are not known when deciding to create a stoma.

The first aim of the study was to perform a retrospective assessment of the impact of LI on the risk of PS and symptomatic AL. The second aim of the study was to assess preoperative PS risk factors in patients with LI.

## Methods

From January 2008 to January 2018, 313 patients with lower rectal cancer underwent surgery at the National Institute of Oncology in Gliwice. Low anterior rectal resection (LAR) was performed with colorectal anastomosis up to 5 cm from the anal verge. In 106 patients, protective LI was performed, and the remaining 207 subjects underwent LAR without LI. The pre-treatment staging was performed based on abdominal and pelvic CT, TRUS and/or pelvic MRI and chest X-ray or CT. All tumours were localised in the lower rectum during the rectal examination. In the radiotherapy (RT) group, the patients received a total dose of 25–42 Gy. In the radiochemotherapy group, the patients received one or two cycles of 5-fluorouracil-based chemotherapy followed by pelvic radiotherapy with a total dose of 42–54 Gy. In the group that received neoadjuvant treatment, two subgroups were additionally distinguished based on whether the time from the completion of RT to surgery was < 6 weeks or ≥ 6 weeks. Before surgery, mechanical bowel preparation was used with oral neomycin. Intravenous perioperative antibiotic prophylaxis was administered. The procedure was performed by laparotomy, and the resection covered the rectum with the mesorectum (TME) up to the level of the pelvic diaphragm, sparing the autonomic nerves. End-to-end anastomosis was performed with a stapler. The integrity of the anastomosis rings was assessed each time, but no air leak test was performed. LI was performed in the right lower abdomen based on the surgeon’s decision in each case.

According to the International Study Group of Rectal Cancer, colorectal AL was defined as a defect in the integrity of the intestinal wall at the colorectal site that leads to a communication between the intra- and extraluminal compartments, as well as pelvic abscesses in the proximity of the anastomosis [10]. AL was considered symptomatic if accompanied by peritonitis on physical examination and/or intestinal pelvic drainage. Before qualification for ileostomy reversal, a water-soluble contrast enema was performed each time to exclude asymptomatic AL. During the closure procedure, intestinal anastomosis was performed side-to-side or end-to-end depending on the surgeon’s preferences. Patients who required adjuvant chemotherapy underwent surgery after adjuvant treatment. Unclosed stoma, regardless of the cause of its creation, was considered PS after 18 months. Part of the analysis was performed after using the propensity score matching (PSM) procedure. The study and control groups were matched in terms of sex, age, body mass index (BMI), Charlson comorbidity index (CCI), pre-treatment clinical stage of the disease and time from radiotherapy to surgery. The patient characteristics before and after PSM are presented in Table 1.

**Table 1:** Patient characteristics before and after the propensity score matching

		Before the propensity score matching				After the propensity score matching			
		Ileostomy (*N* = 101)	No ileostomy (*N* = 185)	Test	*p* value	Ileostomy (*N* = 93)	No ileostomy (*N* = 93)	Test	*p* value
Sex	Female	33.7% (*N* = 34)	39.5% (*N* = 73)	Chi-square	0.4007	36.6% (*N* = 34)	35.5% (*N* = 33)	Chi-square	1
	Male	66.3% (*N* = 67)	60.5% (*N* = 112)			63.4% (*N* = 59)	64.5% (*N* = 60)		
Age	*N*	101	185	Mann-Whitney *U*	0.0708	93	93	Mann-Whitney *U*	0.6256
	Mean (SD)	61.98 (10.6)	63.96 (10.75)			62.69 (10.26)	62.62 (11.17)		
	Median (IQR)	61 (55–71)	65 (58–72)			62 (56–71)	64 (59–69)		
	Range	29–83	26–88			29–83	26–88		
BMI	*N*	101	185	Mann-Whitney *U*	0.3901	93	93	Mann-Whitney *U*	0.6423
	Mean (SD)	26.86 (4.41)	26.32 (4.33)			26.63 (4.42)	26.89 (4.53)		
	Median(IQR)	26 (23.8–29.7)	25,8 (23.4–28.4)			25.8 (23.8–29)	25.7 (23.9–29.9)		
	Range	18.4–42.6	14.8–42.5			18.4–42.6	14.8–42.5		
CCI	0	69.3% (*N* = 70)	65.4% (*N* = 121)	Chi-square	0.7288	69.9% (*N* = 65)	66.7% (*N* = 62)	Fisher	0.8288
	1–5	23.8% (*N* = 24)	28.1% (*N* = 52)			24.7% (*N* = 23)	25.8% (*N* = 24)		
	> 5	6.9% (*N* = 7)	6.5% (*N* = 12)			5.4% (*N* = 5)	7.5% (*N* = 7)		
Clinical stage	II	29.7% (*N* = 30)	35.7% (*N* = 66)	Chi-square	0.5922	32.3% (*N* = 30)	31.2% (*N* = 29)	Fisher	0.8885
	III	63.4% (*N* = 64)	57.8% (*N* = 107)			62.4% (*N* = 58)	61.3% (*N* = 57)		
	IV	6.9% (*N* = 7)	6.5% (*N* = 12)			5.4% (*N* = 5)	7.5% (*N* = 7)		
RT	No RT	20.8% (*N* = 21)	17.3% (*N* = 32)	Chi-square	0.0797	21.5% (*N* = 20)	18.3% (*N* = 17)	Chi-square	0.7094
	Time RT-OPER > 6 weeks	50.5% (*N* = 51)	40.5% (*N* = 75)			47.3% (*N* = 44)	45.2% (*N* = 42)		
	Time RT-OPER < 6 weeks	28.7% (*N* = 29)	42.2% (*N* = 78)			31.2% (*N* = 29)	36.6% (*N* = 34)		
PNI	*N*	101	185	Mann-Whitney *U*	0.9463	93	93	Mann-Whitney *U*	0.7634
	Mean (SD)	41.41 (4.14)	41.34 (4.05)			41.44 (4.22)	41.54 (4.13)		
	Median (IQR)	41.7 (39.8–43.7)	41.7 (39.4–43.6)			41.7 (39.8–43.7)	42 (39.4–43.8)		
	Range	18.4–51	28.4–50.7			18.4–51	28.4–49.7		
Sympt AL	Yes	5% (*N* = 5)	23.8% (*N* = 44)	Fisher	< 0.001	5.4% (*N* = 5)	20.4% (*N* = 19)	Fisher	0.0037
	No	95% (*N* = 96)	76.2% (*N* = 141)			94.6% *(N* = 88)	79.6% (*N* = 74)		

*SD* standard deviation, *IQR* interquartile range, *BMI* body mass index, *CCI* Charlson comorbidity index, *RT* radiotherapy, *Time RT-OPER* time from completion of radiotherapy to operation, *Sympt AL* symptomatic anastomotic leakage, *PNI* Prognostic Nutritional Index

Risk factors for PS were assessed in patients with LI. Among the potential risk factors the following were analysed: sex, age, BMI, body surface area (BSA), presence of comorbidities expressed on the CCI scale, coronary artery disease (CAD), diabetes mellitus (DM), pre-treatment stage of the disease, neoadjuvant treatment, time from completion of radiotherapy to surgery, preoperative values of peripheral blood morphotic elements, neutrophil-to-lymphocyte ratio (NLR), platelet-to-lymphocyte ratio (PLR), preoperative fibrinogen plasma concentration (FIBR) and the Prognostic Nutritional Index (PNI).

Percentage distributions (for variables on a nominal scale) or descriptive statistics, including the mean, median, standard deviation (SD) and first and third quartile values (Q1-Q3), are used to describe patient characteristics, complications and treatment efficacy on different scales. The nonparametric Mann-Whitney *U* test was used to compare numerical variables between two groups of observations. The Fisher’s test or the chi-square test was used to investigate the relationship between two categorical variables. A logistic regression model was constructed by selecting a subset of variables based on the univariate analysis, followed by backward stepwise elimination using the Akaike information criterion (AIC). An analysis was performed to clarify whether a fibrinogen-based test could be useful for differentiating patients requiring PS. For this purpose, a receiver operating characteristic (ROC) curve was plotted, for which the optimal threshold was selected, and the sensitivity and specificity of the method were determined based on this optimal point. All calculations were made using the statistical package R version 3.6.0

## Results

A total of 233 (81.5%) patients underwent neoadjuvant treatment (radiotherapy or radiochemotherapy). In the LI group, 1 (0.9%) patient was excluded from the analysis due to death in the postoperative 30-day period. In addition, 4 patients in whom an ileostomy was created due to technical problems during the formation of the anastomosis were excluded from the LI group. In the group of patients without LI, 2 (1%) deaths occurred in the postoperative 30-day course, and these patients were excluded from further analysis. In this group, we also excluded 2 patients in whom intraoperative technical problems during anastomosis creation were found. To avoid overlooking late leakages, 18 patients without AL with the follow-up of less than 3 months were excluded from the non-LI group. Finally, 286 patients were analysed. A total of 101 (35.3%) patients underwent LAR with LI (study group A), while 185 (64.7%) subjects underwent LAR without LI (control group B). The mean follow-up in group A was 46 months (median 41 months) and 50 months in group B (median 43 months). Symptomatic AL was significantly more prevalent in group B compared to group A (*p* < 0.001, Fisher’s test). In group B, symptomatic AL occurred in 44/185 (23.8%) patients, while in group A, symptomatic AL was observed in 5/101 (5%) patients. After PSM, symptomatic AL was found in 20.4% and 5.4% of patients in groups B and A, respectively (*p* < 0.01). In group A, in 3 patients with symptomatic AL, the colorectal anastomosis had to be separated, and Hartmann’s procedure was performed. Of the remaining 98 patients, 32 (32.7%) were not qualified for LI closure. The causes are presented in Table 2. Of the 13 patients whose stoma was not closed due to dissemination/progression of the disease, 5 presented with synchronous liver metastases. One of these patients underwent simultaneous metastasectomy, while the other four had potentially resectable metastases. Ileostomy was closed in 66 patients. Four (6%) of these patients had postoperative complications grade 3B and 1 (1.5%) had grade 4 complications according the Clavien classification system. As a result of the complications, 2 of these patients had PS. In addition, there was 1 (1.5%) death in the postoperative 30-day period. This case was also classified as PS. The median time from the primary surgery to closure of the ileostomy was 8 months (range 2–18 months). In group B, among the patients with symptomatic leakage, it was necessary to separate the anastomosis and perform Hartmann’s procedure in 34 cases. In the remaining 10 cases, the anastomosis was preserved, and a protective stoma was created, which was then closed in 5 cases. Finally, in group A, PS was found in 38/101 (37.6%) patients, while in group B, PS was found in 39/185 (21.1%) patients (*p* < 0.01, chi-square test). After PSM was conducted in the study and control groups, PS was found in 36.6% and 17.2% of cases, respectively (*p* < 0.01, chi-square test).

**Table 2:** Causes of non-closure of ileostomy

	*N*	%
Progression of the disease	13	40.6
Patient refusal	6	18.7
Anastomotic leak	5	15.6
Death during the observation	3	9.4
No eligibility for anaesthesia	2	6.3
Local recurrence	2	6.3
Anastomotic stricture	1	3.1
Total	32	100.0

**Fig. 1:**
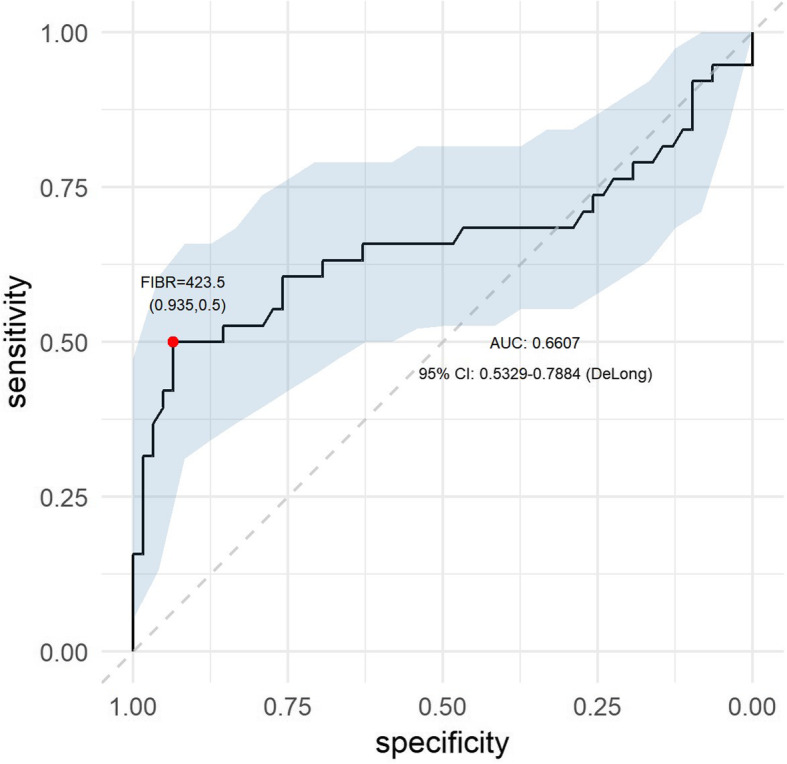
ROC curve of preoperative fibrinogen plasma concentration as a predictor of permanent stoma

Assessment of preoperative risk factors for PS in a group of patients with ileostomy (group A) was performed. The results of the univariate analysis are presented in Table 3. Higher values on the CCI scale were associated with the occurrence of PS (*p* < 0.01, Mann-Whitney *U* test) due to distant metastases. The pre-treatment clinical stage was significantly associated with the risk of PS (*p* < 0.001, Fisher’s test). All patients with distant metastases at the time of surgery had PS. The mean FIBR was 400.08 mg/dl in the group of patients with PS and 342.53 mg/dl in the other patients (*p* < 0.01, Mann-Whitney *U* test). The other analysed risk factors were insignificant in the univariate analysis. Based on the above analysis and the backward stepwise method, a logistic regression model was constructed. FIBR and CCI values were adopted as potential predictors of the risk of PS. The latter was discarded during variable selection by backward elimination. All other factors being equal, each unit increase in the FIBR value translated into a 1.007 higher risk of PS (*p* = 0.013) (Table 4). Due to the abovementioned statistical dependency between variables, the sensitivity and specificity of the hypothetical diagnostic test to assess the risk for PS based on fibrinogen values were calculated. For this purpose, a ROC curve was plotted to estimate the optimal cutoff fibrinogen value, which was 423.5 mg/dl. At this value, the specificity of the method was 0.935, while its sensitivity was 0.5. The positive predictive value (PPV) of fibrinogen values for predicting PS was 82.6%, while the negative predictive value (NPV) was 75.3%. The area under curve parameter (AUC) was approximately 0.6607. A 95% confidence interval was estimated based on DeLong’s method and ranged from 0.5329 to 0.7884. (Fig. 1) This means that the AUC value was significantly higher than 0.5. Hence, we can assume that dividing patients based on the estimated cutoff FIBR value is significantly closer to the actual division of patients with PS than a random division of patients.

**Table 3:** Univariate analysis of preoperative risk factors for permanent stoma in a group of ileostomy patients (group A)

			Permanent stoma			
		Total (*N* = 101)	Yes (*N* = 38)	No (*N* = 63)	Test	*p* value
Sex	Female	33.7% (*N* = 34)	21.1% (*N* = 8)	41.3% (*N* = 26)	Chi-square	0.0621
	Male	66.3% (*N* = 67)	78.9% (*N* = 30)	58.7% (*N* = 37)		
Age	*N*	101	38	63	Mann-Whitney *U*	0.5722
	Mean (SD)	61.98 (10.6)	61.61 (10.39)	62.21 (10.81)		
	Median (IQR)	61 (55–71)	60 (55–71.75)	62 (56.5–69)		
	Range	29–83	40–82	29–83		
Age ≥ 65	Yes	41.6% (*N* = 42)	36.8% (*N* = 14)	44.4% (*N* = 28)	Chi-square	0.5874
	No	58.4% (*N* = 59)	63.2% (*N* = 24)	55.6% (*N* = 35)		
BMI	*N*	101	38	63	Mann-Whitney *U*	0.4241
	Mean (SD)	26.86 (4.41)	26.61 (4.75)	27.02 (4.23)		
	Median (IQR)	26 (23.8–29.7)	25.75 (23.1–29.38)	26.1 (24.2–29.75)		
	Range	18.4–42.6	18.4–38.7	19.4–42.6		
BMI ≥ 30	Yes	23.8% (*N* = 24)	23.7% (*N* = 9)	23.8% (*N* = 15)	Chi-square	1
	No	76.2% (*N* = 77)	76.3% (*N* = 29)	76.2% (*N* = 48)		
BSA	*N*	101	38	63	Mann-Whitney *U*	0.3732
	Mean (SD)	1.87 (0.22)	1.9 (0.24)	1.86 (0.21)		
	Median (IQR)	1.88 (1.71–2.04)	1.9 (1.7–2.08)	1.85 (1.72–1.99)		
	Range	1.32 – 2.42	1.4 – 2.42	1.32 – 2.38		
CAD	Yes	6.9% (*N* = 7)	10.5% (*N* = 4)	4.8% (*N* = 3)	Fisher	0.4206
	No	93.1% (*N* = 94)	89.5% (*N* = 34)	95.2% (*N* = 60)		
HA	Yes	37.6% (*N* = 38)	39.5% (*N* = 15)	36.5% (*N* = 23)	Chi-square	0.9314
	No	62.4% (*N* = 63)	60.5% (*N* = 23)	63.5% (*N* = 40)		
DM	Yes	12.9% (*N* = 13)	15.8% (*N* = 6)	11.1% (*N* = 7)	Chi-square	0.7088
	No	87.1% (*N* = 88)	84.2% (*N* = 32)	88.9% (*N* = 56)		
CCI	0	69.3% (*N* = 70)	52.6%(*N* = 20)	79.4% (*N* = 50)	Fisher	< 0.001
	1–5	23.8% (*N* = 24)	28.9% (*N* = 11)	20.6% (*N* = 13)		
	> 5	6.9% (*N* = 7)	18.4% (*N* = 7)	0% (*N* = 0)		
Clinical stage	IV	6.9% (*N* = 7)	18.4% (*N* = 7)	0% (*N* = 0)	Fisher	< 0.001
	I–III	93.1% (*N* = 94)	81.6%(*N* = 31)	100% (*N* = 63)		
Time RT-OPER	No RT	20.8% (*N* = 21)	18.4% (*N* = 7)	22.2% (*N* = 14)	Chi-square	0.374
	≥ 6 weeks	50.5% (*N* = 51)	44.7% (*N* = 17)	54% (*N* = 34)		
	< 6 weeks	28.7% *(N* = 29)	36.8% (*N* = 14)	23.8% (*N* = 15)		
FIBR	*N*	100	38	63	Mann-Whitney *U*	0.0073
	Mean (SD)	364.4 (88.05)	400.08 (116.1)	342.53 (55.93)		
	Median (IQR)	351 (301.75–419.25)	410.5 (301.5–467.75)	341 (303.75–375)		
	Range	212–721	212–721	242–491		
NLR	*N*	101	38	63	Mann-Whitney *U*	0.2119
	Mean (SD)	3.88 (2.06)	4.33 (2.45)	3.61 (1.75)		
	Median (IQR)	3.3 (2.5–5.1)	3.5 (2.6–5.8)	3.1 (2.45–4.45)		
	Range	1–12.2	1–12.2	1 – 9.9		
PLR	*N*	101	38	63	Mann-Whitney *U*	0.8499
	Mean (SD)	262.2 (141.13)	269.6 (174.16)	257.73 (118.2)		
	Median (IQR)	232.9 (161.9–337.9)	244.45 (143.93–351.9)	232.9 (164.25–335.15)		
	Range	49.3–918.8	49.3–918.8	74.2–509.5		
PNI	*N*	101	38	63	Mann-Whitney *U*	0.1567
	Mean (SD)	41.41 (4.14)	40.41 (4.74)	42.01 (3.63)		
	Median (IQR)	41.7 (39.8–43.7)	41.7 (38.52–43.12)	41.7 (39.9–44.15)		
	Range	18.4–51	18.4–47.9	28.3–51		

*SD* standard deviation, *IQR* interquartile range, *BMI* body mass index, *BSA* body surface area, *CAD* coronary artery disease, *DM* diabetes mellitus, *HA* arterial hypertension, *CCI* Charlson comorbidity index, *Time RT-OPER* time from completion of radiotherapy to operation, *FIBR* preoperative plasma fibrinogen concentration [mg/dl], *NLR* neutrophil-to-lymphocyte ratio, *PLT* platelet-to-lymphocyte ratio, *PNI* Prognostic Nutritional Index

**Table 4:** Logistic regression model explaining the risk of getting a permanent stoma in ileostomy patients

	Odds ratio	2.5%	97.5%	Pr(>|z|)
FIBR	1.007	1.002	1.013	0.013
PNI	0.944	0.831	1.055	0.335
No RT	Ref.			
Time RT-OPER ≥ 6 weeks	0.784	0.245	2.585	0.681
Time RT-OPER < 6 weeks	1.493	0.43	5.388	0.53

*FIBR* preoperative plasma fibrinogen concentration [mg/dl], *PNI* Prognostic Nutritional Index, *RT* radiotherapy, *Time RT-OPER* time from completion of radiotherapy to operation

## Discussion

LI significantly reduced the risk of symptomatic AL, which is in line with other authors [11, 12]. However, LI was associated with a significantly higher risk of PS before and after PSM. In more than one-third of patients, the temporary LI was not closed after 18 months. Thus, the success of sphincter-saving surgical treatment is questionable. One of the reasons is probably our strategy to reverse ileostomy after the completion of adjuvant treatment. We assumed that our priority was the completion of oncological treatment. The optimal time to close the protective ileostomy after LAR has not been clearly determined yet. Early (8–13 days) reversal procedures have been shown to be safe in selected patients [13, 14]. However, there are reports that both early (< 30 days) and late (> 6 months) stoma closure may be associated with an increased rate of postoperative complications [15, 16]. Despite several reports of good functional and oncological results of stoma reversal during adjuvant chemotherapy, there have been no known results of randomised trials to date [17, 18]. The occurrence of postoperative complications related to the closure of the stoma before or during adjuvant chemotherapy may be a reason to discontinue oncological treatment.

The prevalence of PS ranges from 9.5 to 33% in other studies. However, the results of most studies cannot be directly compared for several reasons. Some authors assessed the prevalence of PS in the whole follow-up [1, 8, 9, 16, 19–21], while others introduced the term “the latest expected time to closure” [2, 3, 22]. Additionally, the assumed latest expected time to closure was different, depending on the author, and even within the same analysis, times were different for patients who received complementary chemotherapy and those who did not [3]. We believe that one of the reasons for the high percentage of PS in our analysis may be our adoption of an 18-month period after which we considered non-closed stoma to be PS. Interesting observations in this respect were provided by Gustafsson et al. who in the same material evaluated the prevalence of PS both during the whole follow-up and after the introduction of the latest expected time to closure and obtained the results of 17.1% and 31.6%, respectively [3]. An additional factor that makes it difficult to compare different analyses is the fact that some authors included in their analyses tumours located not only in the lower rectum. In the assessment of PS risk factors in the group of patients with LI (group A), only preoperative and intraoperative factors were considered, since only these factors are useful when making a decision about creating LI. Postoperative factors described in the literature, such as AL, local recurrence or metachronous dissemination, cannot be used in the decision-making model [19, 21, 23]. In addition, we did not distinguish between primary (no qualifications for stoma closure) and secondary PS risk factors (complications after the reversal procedure). However, we searched for factors independent of the aetiology of PS. We did not confirm the impact of some of the PS risk factors described, such as age or the presence of comorbidities [24]. CCI proved to be an important factor in the univariate analysis, but only at > 5, which was associated with the presence of synchronous distant metastases. The analysis did not show, however, the impact of specific diseases such as CAD or DM on PS risk. It seems that stage IV patients are not suitable candidates for LAR with LI, even in cases where the metastases are potentially resectable.

The only risk factor for PS in group A in the multivariate analysis was preoperative fibrinogen concentration. The obtained predictive values for preoperative fibrinogen concentration were insufficient for this variable to be considered an independent factor in the preoperative assessment of PS risk. However, the obtained PPV and NPV of 82.6% and 75.3%, respectively, indicated that the preoperative fibrinogen concentration may be useful in the decision-making model.

It has been shown that a high plasma fibrinogen concentration is associated with the development and progression of tumours. Although this phenomenon has not been fully understood yet, the hypotheses include the ability of fibrinogen to bind growth factors and tumour cells or affect the escalation of the inflammatory response in the tumour environment. These mechanisms lead to the proliferation of cancer cells and increase their invasive potential [25]. Fibrinogen concentration before treatment is a known prognostic factor not only in primary and metastatic colorectal cancer but also in other gastrointestinal cancers in terms of overall and disease-free survival [26, 27]. Additionally, the concentration of fibrinogen, which is also an acute phase reactant, is increased during the intense inflammatory response. It was also shown that presurgical systemic inflammatory response increases the risk of infectious postoperative complications, which also include AL [28–30]. In our material, 72% of the causes of non-closure of the stoma (Table 2) were related to the progression of the disease, local recurrence, death or anastomotic leakage. All these conditions are associated with an increased fibrinogen concentration. As a result, this factor was the only independent predictive factor of PS risk in the multifactorial analysis. At the same time, the analysis showed that above the cutoff point (fibrinogen concentration above 423.5 mg/dl), PS was found in 82% of patients in the group of patients with LI.

Obviously, these are preliminary observations that need to be confirmed and validated in other independent groups of patients with a protective ileostomy in order to confirm the importance of the elevated fibrinogen concentration as a factor increasing the risk of PS, which is known at the time of the decision to create an ileostomy.

Nonetheless, we have not shown the impact of the pre-treatment clinical stage of the disease, other than stage IV, on the risk of PS, although the stage of the disease is an important risk factor for metachronous metastases and survival [31].

It is important to note a relatively high percentage of patients who did not agree to ileostomy reversal. It should be assumed that these patients were insufficiently informed about the treatment method or did not understand the information provided. This indicates the need to pay special attention to informing patients about the consequences of creating LI, including information about the risk of PS. In some of these patients, although sphincter preservation was technically possible, an abdominoperineal resection or low Hartmann’s procedure with a permanent colostomy should be considered, as these procedures have a lower risk of postoperative complications. Colostomy would be more beneficial than a permanent ileostomy [21, 32–34]. When discussing the treatment plan, we need to ensure that the patient understands all the possible consequences of a diverting stoma. It should also be considered that patients with a higher education level are more likely to quickly undergo ileostomy closure [3]. In the absence of randomised studies on the impact of ileostomy closure time on long-term oncological results, we prefer to close the ileostomy only after adjuvant treatment. Furthermore, our analysis indicates a significant difficulty in performing a preoperative risk assessment of PS.

The analysis has the limitations typical of retrospective analyses. The decision to perform an ileostomy was always made subjectively by the surgeon. When analysing the percentage of protective ileostomies performed in the following years, there was a clear trend towards stoma creation. It was not possible to assess the total percentage of ALs, and only symptomatic ALs were detected because in patients without an ileostomy, radiological examinations with a contrast enema were performed only in cases of suspected AL. Additionally, in the ileostomy group, radiological examinations were not performed in 11% of the patients. In our study, the percentage of symptomatic ALs in patients without an ileostomy may be biased since patients with a short follow-up were excluded from the analysis.

## Conclusions

Although protective LI may reduce the incidence of symptomatic AL, it can be related to a higher risk of PS in this group of patients. The plasma fibrinogen concentration before LAR can be a risk factor for PS in LI patients and may be a useful variable in decision-making models.

## Supplementary information

**Additional file 1.**

## Data Availability

All data generated or analysed during this study are included in this published article [and its supplementary information files].

## Abbreviations

AIC: Akaike information criterion
AL: Anastomotic leakage
AUC: Area under the curve
BMI: Body mass index
CAD: Coronary artery disease
CCI: Charlson comorbidity index
CI: Confidence interval
CT: Computed tomography
DM: Diabetes mellitus
FIBR: Fibrinogen
IQR: Interquartile range
LAR: Low anterior resection
LI: Loop ileostomy
MRI: Magnetic resonance imaging
NLR: Neutrophil-to-lymphocyte ratio
NPV: Negative predictive value
OR: Odds ratio
PLR: Platelet-to-lymphocyte ratio
PNI: Prognostic Nutritional Index
PPV: Positive predictive value
PS: Permanent stoma
PSM: Propensity score matching
ROC: Receiver operating characteristic
RT: Radiotherapy
SD: Standard deviation
TME: Total mesorectal excision
TRUS: Transrectal ultrasonography
